# Supplementary material for: RNA-Seq is not required to determine stable reference genes for qPCR normalization
Source: PLoS Comput Biol. 2022 Feb 28;18(2):e1009868. doi: 10.1371/journal.pcbi.1009868 (PMC8912902; doi:10.1371/journal.pcbi.1009868)
Supplement: Attachment — Submitted filename: 120034_1_rebuttal_2223684_r2hfwn.pdf [file pcbi.1009868.s005.pdf]

Université de Paris

Dr. Venkat Krishnan SUNDARAM
INSERM U1124 - Unit T3S
Team 3: Myelination and Nervous System Pathologies
Faculty of Basic and Biomedical Sciences
Université de Paris
45, Rue des Saints Pères 75006 Paris
Email: venkatkrishnan.sundaram@u-paris.fr

Reviewers
Review Commons

Paris, 12th November 2021

Dear Reviewers,

We have addressed all the comments raised and we have made the required changes to the manuscript as suggested. The revised manuscript has been submitted with “tracked changes” as a word document for your reference.

We thank you all for your time and efforts in evaluating our manuscript through the *Review Commons* platform and we hope that we have amply addressed all your concerns.

Sincerely and on behalf of all authors,

Venkat Krishnan SUNDARAM
Co-corresponding author

Author Response to Reviewer 1:

1. We have indeed calculated primer efficiencies by serial dilution and performed a four-point standard curve wherever possible. In other cases, at least a three point dilution curve was performed to assess primer efficiency. To have a more extensive range of Cq values in the standard curve, the dilution series was done with serial dilution by a factor of $1/10^{th}$ as indicated in the materials and methods section under the heading "Amplification Efficiencies". This provided a range of 6.6 cycles (three point dilution) and about 9.9 cycles (4 point dilution for the primers tested. If the Cq values of the 4th dilution fell beyond the detection range of the machines (above 29 cycles) or closer to the No-RT Control, only the first three dilutions were taken into consideration. We have now included the standard curve for all the genes in the metadata/source data and updated the Figshare DOI. All sample Cq values were within this standard curve as mentioned in the materials and methods section and they have been disclosed already in the metadata files. The raw qPCR Cq output for all references and targets for both datasets can be retrieved from the data file in figshare. Moreover, we will also add a new sentence in the methods sections clarifying the standard curve dilutions and data availability.

2. We apologize for any lack of clarity on this line. We have always compared the Control group to the Test group while performing statistical analysis as shown Figure 3 and Figure 6. This is a fundamental point of any study and we have strictly adhered to this. The highlighted statement pertains to the supplementary figures 1 & 2 where we compare the Fold changes of the "Test" groups between qPCR and RNA-Seq in both datasets. Comparing the Control groups with one another between these methods is redundant as the mean Fold change of the control groups are always 1 as we are measuring relative expression. Thus, we cannot perform any meaningful statistical testing between the control groups between RNA-Seq and qPCR regardless of the method employed for testing.

   Furthermore, the use of the $2^{-\Delta\Delta Ct}$ method for relative expression is in strict adherence to the initial papers describing this method (Livak and Schmittgen 2001, Schmittgen and Livak, 2008), which is again recapitulated in the article that you have cited. This can be seen in the metadata where the excel files that were used for calculating differential expression for all samples and datasets can be accessed. However, we would like to remark that we use more stringent criteria for primer validation (Efficiency between 95% and 105% as opposed to between 90% and 110% as mentioned in the paper). Moreover, the statistical testing and data representation prescribed in Figure 5 of the article that you have mentioned are not well founded for the following reasons:

   - We cannot perform parametric T-Tests using low sample sizes. Furthermore, we cannot test for data normality using few data points employed in standard qPCR assays. Thus, neither our qPCR assays nor the ones used in the mentioned article have enough samples to perform a T-Test. Hence, we have used a non – parametric ordinal Mann Whitney test for testing statistical significance in our study, as it is more apt for such low sample sizes and distributions.

   - The article proposes data representation with the mean and SEM or 95% Confidence Intervals (CI). We would like to kindly remark that SEM and CI are sampling parameters that arise when we perform sampling of data points from a larger population. In our study, we have always shown all the data points (biological replicates) for each experimental group. Hence, we can only show the distribution around the mean with the standard deviations (SD) and not with SEM or CI. We have not performed any sampling whatsoever nor has the study mentioned by the reviewer.

3. The method used in our study for reference gene validation is a combination of CV, Normfinder and statistical testing of raw expression profiles. In our previous study (Sundaram et al 2019), we have categorically shown that using a combination of different existing methods such as GeNORM, NormFinder and Best Keeper and comparing their ranks results in a sub optimal choice of reference genes. This is because GeNorm ranks genes with similar expression patterns as stable even if they vary significantly among groups. BestKeeper calculates variation based on Cq values which are exponents while the expression levels are calculated in the linear scale (2^Cq). NormFinder stability scores are influenced by the presence of genes with significant overall variation. More evidence backed up with data can be found in our previous study where we have clearly shown that combining these methods and calculating an overall rank (as proposed in the article you have mentioned) is not the best strategy. Hence, we devised the approach used in the present study, which has been previously validated, published (Sundaram et al 2019, PLoS ONE) and was designed taking into account the advantages and disadvantages of the different existing approaches.

4. The target genes used in the study range from about 12 cycles to about 29 cycles (both datasets included, please refer to the source data/metadata). This falls well within the standard curves of all these genes used as mentioned earlier. The stability of the reference genes has been shown with absolute parameters such as the Co-efficient of variation and the Normfinder S scores (Tables 1, 2, 3 & 4). Although we are not opposed to adding more target genes, we fail to see as to how adding target genes with Cq values above 29 cycles would reflect on the stability of reference genes. The variability that will be observed is a mere reflection of the variability of Cq values of the target genes in the Cq range of 29 – 32 as it approaches the detection limits of qPCR assays. The Cq values of the best reference genes would still remain the same. Therefore, this exercise cannot test the "stability" of the reference genes but only demonstrate the limit of qPCR detection (which is already well known). We would also like to remark that we have used No-RT controls in our qPCR assays, which exhibit a signal (different dissociation peak) in this Cq range for some genes and hence this is not a signal that arises from the cDNA. Therefore, we do not consider values above 29 cycles are reliable in our qPCR setup and we switch to droplet digital PCR for such low-expressed genes in our studies.

Author Response to Reviewer 2:

We understand the reviewer's perspective on the use of the words "random reference genes". We have replaced it with the words "conventional reference genes" throughout the manuscript.

Regarding the addition of other RNA species as reference genes, we would like to clarify that we have used only protein coding transcripts (encoded by nuclear genes) as reference genes as all our target genes also belong to the same RNA category. This was done in accordance with the MIQE guidelines for qPCR data publication (Bustin et al 2009, DOI: 10.1373/clinchem.2008.112797) which states that rRNA should not be used for mRNA target gene normalization. This is because the vast majority of RNA from total RNA extraction is rRNA and only about 1% - 5% is mRNA. Thus, it is advisable to normalize mRNA targets with mRNA reference genes as it serves as a control for the extraction and RT PCR protocol. This argument can also be extended to other RNA species either in type or in origin (mtRNA). Regarding the total RNA factor, we have always used the same quantity of total RNA from all samples for RT-PCR as mentioned in the materials and methods section.

Author Response to Reviewer 3:

All minor concerns have been addressed in the revised manuscript as follows:

1. Figure 1, 2, 3, 4, 5, 6: in the figure are uppercase letters, in the figure legend are lowercase letters, please adjust that – **Has been modified**
2. p10 line 347: I understand what is meant, with "using the NF as the reference gene", however, stating again that the combined NF of the two most stable ref genes was used here, would make it clearer. – **Has been modified**
3. P11 line 355f: the first sentences here are negligible, as already stated elsewhere – **Have been removed**
4. P30 line 777: The last sentence is not clear to me.

   We wanted to say that our study aptly addressed the strongest hurdle in performing reliable qPCR assays, which is the choice of good reference genes. This choice is not dependent on RNA-SEQ results. We have modified this sentence for better clarity.
